# Predicting Time to Take-Off in a Countermovement Jump for Maximal Quickness from Upright and Squat Starting Positions

**DOI:** 10.2478/hukin-2022-0091

**Published:** 2022-11-08

**Authors:** Tal Amasay, David N. Suprak

**Affiliations:** 1 Barry University, Miami Shores, FL, USA.; 2 Western Washington University, Bellingham, WA, USA.

**Keywords:** rate of force development, impulse, countermovement, jump time, force

## Abstract

A countermovement jump (CMJ) is common in sport and often time-constrained. Little is known about contributors to quickness in jumps. This study examined effective predictors of time to take-off and effects of the starting position on reaction time and take-off time in a countermovement jump performed for quickness from upright and squat positions. Forty-nine collegiate athletes performed CMJs for quickness from upright and squatting starting positions to 75% of their maximal jump height. Several variables were calculated from the kinetic data related to jump performance. Correlation and multiple regression were used to determine variables related and predictive of time to take-off under both conditions. Paired t-tests evaluated differences in reaction and take-off times between conditions. In the upright condition, an increasing rate of force development and force, and decreasing time variables, impulses, and countermovement depth were associated with shorter time to take-off. The time to take-off prediction included rates of force development, force, time, and impulse. In the squat condition, shorter time to take-off was associated with lesser time variables, eccentric impulse, force at the end of the eccentric phase, and countermovement depth, and a greater rate of force development, concentric impulse, peak power, peak force, and reaction time. The time to take-off prediction equation included time to the bottom of the countermovement, time to peak force, and peak power. Reaction and take-off times were longer in the upright condition. Quick jump efficiency may be improved by strategies to increase maximum strength and the eccentric rate of force development while decreasing countermovement depth and time to bottom.

## Introduction

A countermovement jump (CMJ) is an explosive jump involving a preliminary downward motion followed by an upward motion, accelerating the center of mass vertically to leave the ground. This type of action is common in many sporting environments and takes advantage of the stretch-shortening cycle (SSC). The SSC is a coupling of the eccentric (lengthening) and concentric (shortening) actions of an agonist muscle to capitalize on the force generation from both the stretch reflex and stored elastic energy in the tendon to maximize force output at the beginning of the concentric phase, resulting in production of net vertical impulse at a higher rate and a shorter amount of time (Guess et al., 2020). The potential of the SSC to result in maximum force output during the concentric phase depends on the range of muscle lengthening, as well as shortening velocity and acceleration (Cormie et al., 2010; Mandic et al., 2015). In addition, a countermovement may allow for development of a higher level of the muscle active state, resulting in greater joint moments at the start of the concentric phase (Bobbert et al., 1996).

The CMJ is used extensively as a simple test that lends insight into the neuromuscular and SSC capabilities of the lower extremity (Perez-Castilla et al., 2020). As such, much work has been done to examine variables related to CMJ execution and their contribution to jump performance and efficiency. CMJ performance is often assessed using 3D camera systems and force plates, specifically examining the vertical ground reaction forces (vGRF) to derive the force-time curve, and numerous related variables. Such research has demonstrated that the countermovement depth (Perez-Castilla et al., 2019; Sanchez-Sixto et al., 2018), the rate of force development in the eccentric phase (ERFD) (Laffaye and Wagner, 2013), peak force (Daugherty et al., 2021; Dowling and Vamos, 1993) and peak power (Barker et al., 2018; Daugherty et al., 2021; Dowling and Vamos, 1993; Harman et al., 1991) during the jump are all positively related to maximal jump height.

In team sport competition, the environment is often dynamic and time-constrained (Barker et al., 2018). In volleyball, for example, vertical jumping is a foundational skill, in which timing is an important constraint, whether it pertains to jumping to spike a ball, or to block an opponent (Domire and Challis, 2015; Lima et al., 2018; Mandic et al., 2016). Therefore, jumps in sport competition are often executed for quickness to submaximal heights from various starting positions. It is, thus, appropriate to incorporate a timing aspect into the evaluation of jump performance (Barker et al., 2018; Domire and Challis, 2015; Siembida et al., 2021). The time to take-off (from the start of the downward movement to toe-off) (TTO) is then a variable of interest in determining contributors to jump timing efficiency. Previous research has indicated that TTO varies with countermovement depth (Mandic et al., 2016). Less is known regarding TTO in comparison to jump height, thus further research regarding its role in evaluating jump performance is warranted.

Previously, Amasay (2008) reported greater maximal jump height when starting from the upright, compared to a self-selected squat, position in a maximal height block jump in collegiate volleyball players. Amasay (2008) also reported longer TTO for block jumps performed for quickness from the upright vs. squat positions, although this difference was not significant. However, little is known regarding the relative importance of factors contributing to success in CMJs performed for maximal quickness from upright and squat starting positions. Therefore, the purpose of the present study was to use a multiple regression approach to determine effective predictors of TTO in a CMJ performed for maximal quickness from upright and squat positions. The secondary purpose was to determine the effects of the starting position (upright vs. squat) on reaction time (RT) and TTO in a CMJ performed for maximal quickness. We hypothesized that time-related variables (including rates of force development) would exhibit greater correlations with, and be more predictive of, TTO than other variables. Furthermore, we hypothesized no significant difference between RT and TTO from the upright and squat positions.

## Methods

### Participants

Forty-nine Division II athletes (22 males) participated in the study. Participant demographics are presented in Table 1. Athletes participating in the study were from different varsity teams such as soccer, basketball, tennis, rowing, softball and baseball. All participants were free of acute injuries prior to testing and cleared by the university sports medicine staff to participate in their team training and this study without limitations. The research protocol was approved by the university institutional review board in accordance with the Helsinki Declaration. All participants read and signed a consent form prior to data collection.

**Table 1:** Participants’ demographics.

	Age (yrs)	Body height (cm)	Body Mass (kg)	College Experience (yrs)	Total Experience (yrs)
Group Mean (SD)	20.2 (1.5)	175.3 (8.6)	73.8 (10.6)	2.8 (1.2)	11.0 (4.6)
Women (SD) Mean	20.4 (1.4)	171.5 (8.2)	67.8 (7.4)	2.9 (1.2)	8.7 (4.6)
Men Mean (SD)	20.0 (1.5)	179.9 (6.6)	81.2 (9.1)	2.7 (1.2)	13.7 (2.7)

### Design and Procedures

All data were collected in a single session. Participants performed a 10-min general and specific dynamic warm-up before starting testing. The general warm-up consisted of riding a stationary bike at a self-selected pace. The specific warm-up consisted of high knees, heel to toes, marching, squats, front lunges, carioca, and submaximal vertical jumps.

Kinetic data were collected using two AMTI force plates (Advanced Mechanical Technology, Inc., Watertown, MA, USA) sampled at 960 Hz, and Vicon Nexus 1.7.1 software (Vicon, Centennial, CO, USA). A low-pass fourth order Butterworth filter with a cutoff frequency of 300 Hz was used to filter all kinetic data. Prior to kinetic testing, a Vertec (Sports Imports, Columbus, OH, USA) was used to record the maximum vertical jump height for each participant to set the target height for quick jumps.

Participants’ body weight was calculated using the summed vGRF from the force plates during a standing trial. Each participant stood on the force plates for at least 3 s. The middle second was used to calculate the average vGRF while standing motionless and the data from the two force plates were summed to calculate participants’ body weight.

Participants performed three maximal height CMJs, from both the upright and squat starting positions. Participants were positioned with one foot on each force plate, and the Vertec positioned so participants could jump vertically and touch its vanes. For the upright maximal jumps, participants began by standing in a comfortable upright position. Participants were instructed to perform a rapid countermovement to a self-selected depth and immediately jump vertically with maximal effort. Participants were required to land with both feet on the force plates on which they began, otherwise jumps were repeated. The maximal CMJ from the squat position was performed identically to the upright jump, but beginning from a self-selected squatting position. Arm movement was not restricted during jumps to encourage natural movement and maximal performance. The highest reach of the three jumps, from each starting position, recorded via the Vertec was taken as their maximal vertical jump. Kinetic data collected during maximal jump trials were used in subsequent analysis.

To perform the quick jumps, the Vertec was set at 75% of participants’ individual maximal jump height recorded previously. The Vertec was positioned so the target vane was located directly over the force plates, and participants could jump and touch it. Each participant performed three quick CMJs to their target height, from upright and squat positions (total of six jumps).

Participants were positioned with one foot on each force plate. A blue diode light was positioned 3 m in front of the participant and 1.5 m above the ground and was used to signal to participants when to begin the jump. When participants indicated they were ready, the investigator began data collection. There was a 2-s delay between the start of data collection and light illumination. Participants were instructed not to anticipate when the light would turn on, but to react to the light as quickly as possible, performing the CMJ to touch the target vane in minimal time. The verbal instruction the participant received before each jump was “when you see the blue light jump as fast as you can to touch the target”. Participants were instructed to jump and land on the force plates, and only those jumps in which this was properly executed were counted. At least 2 min rest intervals were afforded between each jump.

**Figure 1:**
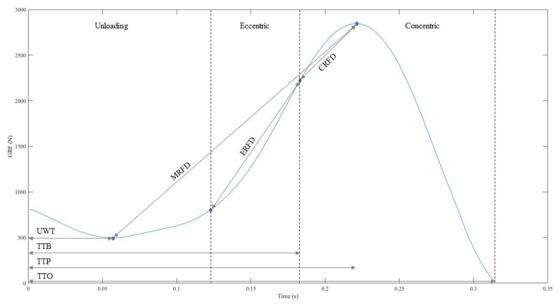
Illustration of jump phases and RFD calculations

Data were analyzed via custom-written Matlab R2020 software (Mathworks, Natick, MA). The start of the countermovement was identified when the vGRF was above or below the body weight by more than 2.5% of body weight (Barker et al., 2018), and stayed for at least 50 data points. Toe-off was identified when the vGRF dropped below 20 N (Barker et al., 2018), and stayed for at least 100 data points. The trial with the quickest TTO was analyzed for each participant for each starting position, upright and squat. Fifteen variables were calculated for each jump, based on the kinetic data collected: TTO (s), eccentric rate of force development (ERFD) (N/s), concentric rate of force development (CRFD) (N/s), mean rate of force development (MRFD) (N/s), peak RFD (PRFD) (N/s), force at the bottom of the countermovement (FAB) (N), peak force (PF) (N), reaction time (RT) (s), unweighting time (UWT) (s), time to bottom of the countermovement (TTB) (s), time to peak force (TTP) (s), eccentric Impulse (EccImp) (Ns), concentric impulse (ConImp) (Ns), peak power (PP) (W/kg), and COM displacement during the countermovement (COMDis) (m). The formulas for each of these variables are shown in Table 2. An illustration of the phases of the CMJ, RFDs and times calculated in this study is presented in Figure 1.

**Table 2:** Variable calculation formulas

Variable	Formula
Peak force (PF) (N)	Maximal vertical GRF during jump
Maximal negative COM displacement (COMDis) (m)	Calculated as double integration of acceleration of the COM. Divides the eccentric (countermovement) and concentric (propulsive) phases.
Force at bottom of the countermovement (FAB) (N)	Vertical GRF at point where COM reaches the maximum negative displacement.
Eccentric rate of force development (ERFD) (N/s)	$ERFD=\frac{FAB-Force\text{ at beginning of eccentric phase }}{\text{ change in time }}$
Concentric rate of force development (CRFD) (N/s)	$CRFD=\frac{\text{ Peak force }-\text{ Force at bottom of countermovement }}{\text{ change tn time }}$
Mean rate of force development (MRFD) (N/s)	$MRFD=\frac{\text{ Peak force - minimum force }}{\text{ change in time }}$
Peak rate of force development (PRFD) (N/s)	Maximum positive slope of vertical GRF over 10-ms intervals
Reaction time (RT) (s)	Elapsed time from when the light turned on to the first movement of the COM (beginning of the jump)
Unweight time (UWT) (s)	Elapsed time from the beginning of the jump to the minimum vertical GRF
Time to bottom of countermovement (TTB) (s)	Elapsed time from the beginning of the jump to the maximal negative COM displacement
Time to peak force (TTP) (s)	Elapsed time from the beginning of the jump to the maximal vertical GRF
Time to take-off (TTO) (s)	Elapsed time from the beginning of the jump to the instant of toe-off, when the subject left the ground
Eccentric impulse (EccImp) (Ns)	The area under the GRF-time curve during the eccentric (countermovement) phase
Concentric impulse (ConImp) (Ns)	The area under the GRF-time curve during the concentric (propulsive) phase
Peak Power Output (PP) (W/kg)	Maximal product of vertical GRF and COM velocity during the jump, normalized to body mass

### Statistical Analysis

IBM SPSS Statistics Version 28 was used for statistical analyses. An a priori power analysis was conducted using pilot data. Based on these pilot data and using the resulting adjusted multiple correlation of *r* = .996, fourteen predictor variables (ERFD, CRFD, MRFD, PRFD, FAB, PF, RT, UWT, TTB, TTP, EccImp, ConImp, PP, and COMDis), and an alpha level of *p* < .05, at least 17 participants would be required to obtain a power level of 0.8.

Pearson correlation coefficients were calculated between each calculated variable and TTO, and their strength was evaluated using benchmarks outlined by Field (2018). Correlation coefficients less than 0.2 were classified as weak, those between 0.2–0.49 were moderate, and those 0.5 and above were strong.

Only significant correlations were used to identify predictor variables to include in the regression analysis. Multicollinearity was evaluated via the variance inflation factor (VIF) for each predictor variable. In the event that two or more predictors exhibited a VIF greater than 10 (Field, 2018), the predictor with the highest bivariate correlation with TTO was kept in the model, while the other predictor was discarded. Once appropriate predictors were identified, they were entered into a backward stepwise multiple regression, with a *t*-test exit criterion of *p* > .1 (Laffaye and Wagner, 2013). The analysis of variance (ANOVA) and adjusted R^2^ were used to evaluate goodness of fit for the resulting regression models for upright and squat conditions.

Paired *t*-tests were conducted to compare RT and TTO across upright and squat conditions. The alpha level was set at *p* < .05. Cohen’s *d* was used to evaluate effect sizes for these comparisons, according to the benchmarks of small (0.2), medium (0.5), and large (0.8) effects (Cohen, 1988).

## Results

### Upright Quick Jump Regression Results

Table 3 shows mean values for each variable calculated in the upright and squat conditions. Several variables exhibited significant correlations with TTO (Table 4). All RFD (ERFD, CRFD, MRFD, and PRFD) and force (FAB, PF) measures were strongly correlated with TTO, as were TTB, TTP, EccImp, and COMDis. UWT and ConImp were both moderately correlated with TTO. No weak correlations were found to be significant.

**Table 3:** Variable mean ± SD by condition

	Upright Quick	Squat Quick
ERFD (N/s)	19,273.30 (10,685.82)	10,281.30 (4833.26)
CRFD (N/s)	7646.60 (5297.52)	6083.14 (3610.38)
MRFD (N/s)	13,305.98 (6261.62)	6150.29 (3212.93)
PRFD (N/s)	35,077.67 (18,572.85)	13,374.02 (6961.68)
FAB (N)	2195.91 (481.60)	972.69 (314.76)
PF (N)	2512.95 (522. 59)	1921.18 (408.30)
RT (s)	0.238 (0.058)	0.196 (0.062)
UWT (s)	0.115 (0.039)	0.062 (0.076)
TTB (s)	0.276 (0.059)	0.107 (0.125)
TTP (s)	0.318 (0.075)	0.297 (0.085)
TTO (s)	0.448 (0.086)	0.415 (0.091)
EccImp (Ns)	67.72 (15.69)	6.76 (10.18)
ConImp (Ns)	179.58 (36.30)	187.95 (41.72)
PP (W/kg)	56.90 (12.02)	52.80 (10.53)
COMDis (m)	0.12 (0.043)	0.009 (0.021)

Variables with significant correlations with TTO were entered into the backward stepwise multiple regression analysis. After excluding variables with non-significant *t*-values, or with VIF greater than 10, variables that remained in the regression model were CRFD, FAB, UWT, TTB, and EccImp (*F*(5, 43) = 534.36, *p* < .001, adjusted *R*^2^ = .982). These variables significantly predicted TTO with the equation: Time to take-off = .147 – 0.000001(CRFD) - 0.000028(FAB) – 0.244(UWT) + 1.283(TTB) + 0.001(EccImp).

### Squat Quick Jump Regression Results

As with the upright quick results, several variables in the squat quick jump were significantly correlated with TTO (Table 5). UWT, TTB, TTP, and EccImp were strongly correlated with TTO. Variables with moderate significant correlations to TTO included ERFD, CRFD, FAB, PF, RT, ConImp, PP, and COMDis. No weak correlations were significant. Variables with significant correlations with TTO were entered into the backward stepwise multiple regression analysis. After excluding variables with non-significant *t*-values, or with VIF greater than 10, variables that remained in the regression model were TTB, TTP, and PP. This model significantly predicted TTO (*F*(3,18) = 165.67, *p* < .001, adjusted *R*^2^ = .959). The resulting prediction equation was: TTO = 0.197 + .228(TTB) + .827(TTP) - .001(PP).

**Table 4:** Pearson’s correlation coefficient for each variable with TTO in the (a) upright and (b) squat starting positions. a.

	ERFD	CRFD	MRFD	PRFD	FAB	PF	RT	UWT	TTB	TTP	EccImp	ConImp	PP	COMDis
TTO	-.725*	-.524*	-.733*	-.565*	-.516*	-.501*	.122	.480*	.966*	.948*	.523*	.283*	-.205	.854*

**Table:** b.

	ERFD	CRFD	MRFD	PRFD	FAB	PF	RT	UWT	TTB	TTP	EccImp	ConImp	PP	COMDis
TTO	-.483*	-.283*	-.221	-.186	.314*	-.391*	-.475*	.740*	.737*	.971*	.551*	-.341*	-.441*	.343*

Pearson’s correlation coefficient for each variable with TTO in the (a) upright and (b) squat starting positions. *Denotes significance (p < .05) #Moderate correlation +Strong correlation

*Upright vs. Squat Condition Comparison*

Paired *t*-tests indicated both RT and TTO were significantly longer in the upright, compared to the squat, quick jump condition (*t*(48) = 3.26, *p* = .002 and *t*(48) = 2.40, *p* = .02, respectively). However, the mean differences were small, with the difference in RT being 0.04 s, and the difference in TTO .033 s. The effect sizes for both comparisons were small (*d* = .469 for RT and *d* = .344 for TTO).

## Discussion

The primary purpose of this study was to determine the most effective predictors of TTO, via bivariate correlation and a multiple regression approach, in a CMJ performed for maximal quickness from both upright and squat starting positions. We hypothesized that time-related variables (including RFD) would exhibit greater correlations with, and be more predictive of, TTO than other variables.

In the quick jump from the upright starting position, our data demonstrated that all RFD (ERFD, CRFD, MRFD, PRFD) and force (FAB and PF) measures were strongly and negatively correlated with TTO, while TTB and TTP were strongly and positively correlated with TTO. In addition, EccImp and COMDis were strongly and positively correlated with TTO. Moderate positive correlations to TTO were uncovered for both UWT and ConImp. Therefore, our data show that an increasing RFD, in both the eccentric and concentric phases, as well as overall (MRFD) and at peak, and force reached at the bottom of the countermovement and at peak, are associated with shorter TTO. Additionally, decreasing time variables (UWT, TTB, TTP), impulses (EccImp and ConImp) and COMDis are associated with shorter TTO. Barker et al. (2018) also reported a strong negative correlation between ERFD and TTO, in a maximal height jump starting from the upright position, although they calculated it from minimum force to FAB. Other authors have previously shown the positive correlation between PF and maximal jump height (Daugherty et al., 2021; Dowling and Vamos, 1993), but to our knowledge, this is the first investigation to show the negative correlation to TTO in a jump executed for quickness.

The significant regression model included such variables as CRFD, FAB, UWT, TTB, and EccImp. Thus, our hypothesis was partially supported regarding the upright starting position, since all RFD and time variables were at least moderately correlated with TTO. However, force variables (FAB and PF) were also strongly correlated with TTO, which did not support our hypothesis.

The regression equation that resulted from our data shows that TTO was best predicted using elements of the RFD (CRFD), force output (FAB), time (UWT and TTB), and impulse (EccImp). According to the present results, ERFD was significantly associated with both FAB (*r* = 0.876, *p* < .001) and TTB (*r* = -.619, *p* < .001). Thus, a higher ERFD may contribute to both shorter TTB and greater FAB, which may result in a more efficient stretch-shortening cycle, and ultimately, shorter TTO (Laffaye and Wagner, 2013). Furthermore, COMDis was significantly correlated with both CRFD (*r* = -.474, *p* < .001) and EccImp (*r* = .661, *p* < .001). This finding may indicate that lesser COMDis depth results in lower EccImp, and therefore, a need for a higher RFD in the concentric phase to project the COM vertically with minimal TTO. These hypotheses should be addressed in future studies since definitive cause-and-effect relationships cannot be established via a correlational study.

In the quick jump from the squat starting position, the current data indicated strong positive correlations of UWT, TTB, TTP, and EccImp with TTO. Moderate positive correlations with TTO were found for FAB and COMDis. ERFD, CRFD, ConImp, PP, PF, and RT were all moderately and negatively correlated with TTO. Therefore, decreasing UWT, TTB, TTP, EccImp, FAB, and COMDis are associated with shorter TTO. Conversely, increasing ERFD, CRFD, ConImp, PP, PF, and RT are all associated with shorter TTO.

The significant regression model predicting TTO in the squat starting position included only the variables TTB, TTP, and PP. Thus, our hypothesis was only partially supported regarding the quick jump from the squat starting position, since the time-related variables and some of the RFD variables were correlated with TTO, but only TTB, TTP, and PP were included in the prediction equation.

In examining the prediction equation, it is evident that lower TTB and TTP, coupled with higher PP, would result in shorter TTO. The positive significant correlations exhibited in the current data for both EccImp (*r* = .838, *p* < .001) and COMDis (*r* = .571, *p* < .001) with TTB may indicate that minimizing COMDis (which is strongly related to EccImp (*r* = -.879, *p* < .001)) in the squat starting position can help minimize TTB, and therefore, TTO. Our data show that PP is strongly related to both PF (*r* = .680, *p* < .001) and ConImp (*r* = .760, *p* < .001). Therefore, to improve the ability to generate PP in the quick jump from the squat position, it may be beneficial to work to increase maximal strength and the RFD (which was also related to PF), thereby improving the capacity to generate high ConImp. Again, these hypotheses should be examined in the future. It is important to note that the mean COMDis and EccImp for the squat quick jump were 0.009 ± 0.021 m and 6.76 ± 10.18 Ns, respectively, with 29 of 49 participants exhibiting no COMDis or the eccentric phase.

Our secondary purpose was to determine the effects of the starting position (upright vs. squat) on RT and TTO in a CMJ performed for maximal quickness. We hypothesized no significant difference between the RTs and TTOs from the upright and squat positions. Our hypothesis was not supported, as RT and TTO were both significantly longer in the upright position. Given the positive correlation between countermovement depth and TTO in the current study in both upright and squat conditions, it makes sense that TTO would be longer in the upright condition, since it takes time to lower the COM from this position into the countermovement to load the lower body and prepare for the concentric phase. This finding is in agreement with the results of Mandic et al. (2016), but contrasts with those of Amasay (2008), who found that TTO was longer in the upright starting position, but the difference was not significant. It is difficult to know for certain the reason for this discrepancy in findings. However, Amasay (2008) studied a more homogenous group of female collegiate volleyball players, who performed only block jumps, rather than using full arm motion. In addition, Amasay (2008) had participants jump to touch a volleyball at a standard height of 2.4 m, in contrast to the height used in this study, which was relative to individual maximal jump height.

Since RT represents the time elapsed from the presentation of the visual stimulus to the beginning of the jump, shorter RT in the squat condition may result from the lower body musculature already being loaded when the stimulus was presented, and thus, less time needed to prepare to begin the jump. This can be advantageous for athletes in sports reacting to the trajectory of a ball or the movement of an opponent.

### Quick Jump Height

The average upright maximum jump height was 0.391 m. The mean target height for the upright quick jumps was, therefore, set at approximately 0.293 m. The average jump height for upright quick jumps was 0.287 m. Therefore, participants tended to undershoot the target jump height in the upright starting position by 0.006 m. However, the target height was set on the Vertec, which has a resolution of 0.013 m (0.5 in) between vanes, thus the degree of undershoot for upright quick jumps was not meaningful.

The average squat maximum jump height was 0.383 m. The target height for the squat quick jumps was, therefore, set at approximately 0.287 m. The average jump height for squat quick jumps was 0.317 m. For that reason, participants tended to overshoot the target jump height in the squat starting position 0.03 m. This overshoot magnitude exceeds the resolution of the Vertec. Thus, participants would have to adjust for this overshoot by repositioning the hand to contact the Vertec vanes. This may indicate that, in the squat condition, participants overestimated the force needed to accelerate the COM vertical to the prescribed height with a smaller (and for many participants, absent) countermovement.

### Limitations

This investigation has several limitations. We did not restrict arm movement during jumping trials. Many authors have cited the reason for restricting arm movement as isolating the contribution of the lower extremity to the jump. We felt that doing so may alter kinematics of the lower extremity and reduce the fluidity characteristic of the more natural jumping movement pattern to which subjects were accustomed, and that the results would be more applicable to sport performance if arm movement was unrestricted.

We did not dictate or regulate neither the depth of the countermovement in the upright condition, nor the starting position in the squat condition. Rather, participants were instructed to use the same countermovement or starting squat position they normally would adopt in their sport. We chose not to alter participants’ preferred squat and countermovement depth in order to render the results more generalizable to the field.

Although our sample consisted of collegiate varsity athletes, they were taken from several different sports, including soccer, basketball, tennis, rowing, softball and baseball. These sports comprise varying demands for jumping and explosive lower extremity movements. Therefore, participants may have had heterogenous skill levels in the movements performed, and this may have affected the results obtained. Including participants from various sporting backgrounds may have limited the specific applicability of the results to those involved in a particular sport. These results may therefore be more appropriately applied to collegiate athletes, in general. In addition, this heterogeneity may have limited the ability to find significant results since variability may have been greater across subjects. The current data still demonstrated significant correlations and regression models that accounted for a large portion of the variability in TTO scores, as well as significant differences in both RT and TTO between conditions. It could be that the effect size of condition on RT and TTO would have been larger with a more homogenous sample, and this should be investigated in the future.

## Conclusion

The force-time derived variables associated with, and predictive of, TTO differ between the CMJ performed for maximal quickness from the upright and squat starting positions. In the upright condition, our data indicated that the increasing RFD and force output, and decreasing time variables, impulses, and countermovement depth were all associated with shorter TTO. The prediction of TTO in the upright condition involved variables CRFD, FAB, UWT, TTB, and EccImp. A higher ERFD may contribute to both shorter TTB and greater FAB, while lesser COMDis depth may lead to lower EccImp, and therefore, a need for a higher RFD in the concentric phase in order to project the COM vertically with minimal TTO. Therefore, given the relation of several measures of the RFD to TTO, and specifically CRFD and FAB to predicting TTO, coaches and practitioners may implement methods such as Olympic weightlifting variations (Haff et al., 2008), resistance training with eccentric phase emphasis (Suchomel et al., 2019), and stretch-shortening cycle exercises (Matavulj et al., 2001) to target improvements in the RFD and FAB, thereby decreasing TTO.

In the squat condition, decreasing time variables, EccImp, FAB, and countermovement depth, and increasing ERFD, CRFD, ConImp, PP, PF, and RT were all associated with shorter TTO. The TTO prediction in the squat condition included TTB, TTP, and PP. Minimizing the COMDis in the squat condition can help minimize TTB, and therefore, TTO. Improving strength and RFD capacity, utilizing methods similar to those described for the upright condition, but adjusted for starting position specificity, may help increase ConImp, and thus, PP. The only variable that helped predict CMJ TTO in both upright and squat starting positions was TTB. Therefore, in sports requiring jumping from a variety of starting positions, it may be advisable to focus attention on strategies aimed at decreasing TTB during the jump by minimizing the countermovement depth (Pérez-Castilla et al., 2020) and maximizing countermovement velocity (Bosco and Komi, 1979). RT was shorter in the squat condition. Coaches and trainers may employ strategies to increase lower extremity musculotendinous loading in athletes in their starting positions to minimize RT.
